# LDP vs ODP for pancreatic adenocarcinoma: a case matched study from a single-institution

**DOI:** 10.1186/s12876-015-0411-2

**Published:** 2015-12-22

**Authors:** Miaozun Zhang, Ren Fang, Yiping Mou, Ronggao Chen, Xiaowu Xu, Renchao Zhang, Jiafei Yan, Weiwei Jin, Harsha Ajoodhea

**Affiliations:** 1 Department of General Surgery, Sir Run Run Shaw Hospital, School of Medicine, Zhejiang University, 3 East Qingchun Road, Hangzhou, 310016 Zhejiang Province China; 2 Department of General Surgery, Zhejiang Provincial People’s Hospital, Wenzhou Medical University, 158 Shangtang Road, Hangzhou, 310014 Zhejiang Province China

**Keywords:** Pancreatic ductal adenocarcinoma, Laparoscopic surgery, Open surgery, Distal pancreatectomy, Case matched study

## Abstract

**Background:**

Laparoscopic distal pancreatectomy (LDP) showed advantage of perioperation outcomes for benign and low-grade tumor of the pancreas. The application of LDP for pancreatic ductal adenocarcinoma (PDAC) didn’t gain popular acceptance and the number of LDP for PDAC remains low. We designed a case-matched study to analysis the short- and long-term outcomes of the patients undergoing either Laparoscopic distal pancreatectomy or open distal pancreatectomy for PDAC.

**Method:**

From 2003 to 2013, 17 patients were underwent LDP and 34 patients were underwent ODP for PDAC were matched by tumor size, age and body mass index (BMI). The two groups’ demographic information, perioperative outcomes and survival data were compared.

**Results:**

Baseline characteristics were comparable between the LDP and ODP groups. The intraoperative blood loss, first flatus, first oral intake and postoperative hospital stay were significantly less in LDP group than ODP group (50 ml vs400ml, *P* = 0.000; 3d vs 4d, *P* = 0.001; 3d vs 4d, *P* = 0.003; 13d vs 15.5d, *P* = 0.022). The mean operation time, overall postoperative morbidity and postoperative pancreatic fistula rates were similar in the two groups. 5 patients (29.4 %) in LDP group and 7 patients (20.6 %) in ODP group underwent extended resections. There were no significant differences in tumor sizes (3.5 cm vs 3.9 cm, *P* = 0.664) and number of harvested lymph nodes (9 vs8 *P* = 0.534). The median overall survival for both groups was 14.0 months. Cox proportional hazards analysis showed extended resections, R1 resection, perineural invasion and tumor differentiation were associated with worse survival.

**Conclusion:**

LDP is technically feasible and safe for PDAC in selected patients and the short-term oncologic outcomes were not inferior to ODP in this small sample study. However the long-term oncologic safety of LDP for PDAC has to be further evaluated by multicenter or randomized controlled trials.

## Background

In the last few decades, with the development of laparoscopic instruments and skills, Laparoscopic distal pancreatectomy (LDP) has become widely accepted by surgeons for benign and low-grade tumors of the pancreas. Recent reviews and meta-analysis showed that LDP has the advantage of less blood loss and fewer hospital stay days as well as fewer postoperative complications compared with open distal pancreatectomy [1–3]. However, application of laparoscopic approach has been restricted for malignant pancreatic lesions due to concerns over oncologic safety [4]. Unlike other gastrointestinal regions, such as stomach and colon, until now, only a few pioneer studies reported direct comparisons of oncologic outcomes between LDP and open distal pancreatectomy (ODP) for pancreatic ductal adenocarcinoma (PDAC) [5–9]. In this study, we designed a 1:2 case-matched retrospective study from a single institution and analysed the short-term and long-term outcomes of the patients undergoing either LDP or ODP for PDAC.

## Methods

### Patient sample and data collection

From April 2003 to December 2013, 68 distal pancreatectomies were performed for PDAC. All the patients were given detailed information about LDP and OPD for PDAC, four experienced surgeons decided the type of operation according to patient’s condition with informed consent. An informed consent was signed by every patient before the study. The exclusion criterias for LDPwere: (1) borderline resectable according to NCCN guidelines [10]; (2) intra-abdominal dissemination; (3) tumor size > 5 cm (located in pancreatic body) or >10 cm (located in pancreatic tail). Invasion of adjacent organs were not considered as contraindications. Cases who underwent laparoscopic exploration before definitive open surgery were not included in either LDP or ODP groups. Finally 17 LDP cases were enrolled in this study and were matched with 34 ODP cases in a 1:2 case-matched design. The patients were matched by three parameters: tumor size (±0.5 cm), age (±5 years), BMI (±1.0). The study was approved by the Institutional Review Board of Sir Run Run Shaw Hosptial of Zhejiang University. All patient data were retrospectively reviewed from cohort database including demographic information, perioperative outcomes and survival data. The following data were collected: gender, age, American Society of Anesthesiologists (ASA), body mass index (BMI), comorbidity, operation time, intraoperative blood loss (EBL), resection margin status, length of hospital stay, postoperative pancreatic fistulae (POPF), postoperative complications, mortality, adjuvant therapy, recurrence, tumor size, tumor differentitation, tumor stage, number of harvested lymph nodes and ration of N1.

### Operative technique

Laparoscopic pancreatic surgery has been carried at our institution since 2003. The first LDP for PDAC was performed in 2004. The standardized technique for LDP at our institution has been previously described [11, 12]. The first 10-mm trocar was inserted below the umbilicus, then the main working trocar (12 mm) and another three assistant trocars (5 mm) were inserted into the right upper flank, left upper flank, left flank, and right flank quadrants respectively; these five trocars were arranged in a V-shape. Briefly, the gastrocolic ligament was divided by a harmonic scalpel (Harmonic Ace; Ethicon Endo-Surgery, Cincinnati, OH, United States) and the lesser sac was entered. Then, the superior border of the pancreas was mobilized and the proximal splenic artery was freed. After mobilization of the inferior border of the pancreas, a retropancreatic tunnel was created under the neck of the pancreas and the pancreas was transected using an endoscopic linear stapler (Endocutter 60 stapler, white or blue cartridge; Ethicon Endo-Surgery, Cincinnati, OH, United States). The splenic artery and the splenic vein were divided at the root. The soft tissue around the common hepatic artery and the celiac trunk were dissected. Then dissection was performed in a “medial – to - lateral” fashion and the distal pancreas along with the spleen were removed. In cases of invasion to adjacent organs such as stomach, left adrenal gland and even left lobe of liver, en bloc resection was performed by laparoscopic approach. The resected specimen was removed using an endoscopic bag by enlarging the incision at the periumbilical port. ODP was performed in a traditional manner or same method as LDP depending on the habit of the surgeon. Frozen section biopsy was applied to ascertain the resection margin.

Postoperative management Oral intake was started after the first flatus passed. The gastric tube was removed immediately after surgery and the urethral catheter was removed on the following day. Patient were discharged if they could tolerate semifluid diet without obvious discomfort and they felt sufficiently recovered without any major complications. Postoperative complications were recorded using the modified Clavien-Dindo classification [13]. Mortality was defined as death occurring during hospitalization or within 30 days. Postoperative pancreatic fistulae were defined as any measurable volume of fluid output (amylase level three times greater than the normal serum level) from drainage tube on or after postoperative day 3 according to the International Study Group on (ISGPF) [14]. R1 resection was considered as tumor extension within 1 mm of margin [15]. TMN stage was applied by the American Joint Committee on Cancer (7^th^ edition). Adjuvant therapy refers to use of motherapy or radiation therapy perioperative or postoperative.

### Patient follow-up

All patients were regularly followed up through outpatient service at the 1, 3, 6, 12 months and 6-month intervals thereafter by telephone call. Recurrences or metastasis were recorded by evidence of imaging examination, laboratory tests or pathologic results from biopsy, cytology or surgical resection. The last follow-up was conducted in Feburary 2015.

### Statistical analysis

Continuous variables were presented as median and range and analyzed using the Student *t* test (parametric distribution) or Mann–Whitney test (nonparametric distribution). Categorical variables were analyzed using Chi Squared and or Fisher’s exact test. Kaplan-Meier method with log rank testing was applied for estimating the survival analysis. Cox proportional hazards analysis was applied to investigate the prognostic factor for overall survival following distal pancreatectomy and variables were entered into the multivariate regression analysis when *P* value was less than 0.2. *P* < 0.05 was considered statistically significant.

## Results

### Baseline characteristics

The baseline characteristics of patients undergoing distal pancreatectomy for PDAC are summarized at Table 1. Seventeen patients underwent LDP while 34 patients underwent ODP. Of the 17 patients in LDP group 2 cases (11.8 %) were converted to open procedure because of local invasion of superior mesenteric artery. The baseline characteristics such as age, gender, BMI, ASA score, comorbidity and ration of extended resection were comparable between LDP and ODP. The most common comorbidity was hypertension and diabetes mellitus (DM).

**Table 1:** Baseline characteristics of patients undergoing distal pancreatectomy for PDAC

Characteristics	LDP (*n* = 17)	ODP (*n* = 34)	*P* value
Age	60 (44–75)	64 (40–76)	0.164
Gender (F)	6 (35.3)	15 (44.1)	0.763
BMI	23.4 (18.7–27.6)	23.7 (19.0–28.7)	0.313
ASA score			0.569
1	9 (52.9)	15 (44.1)	
2	8 (47.1)	19 (55.9)	
Comorbidity	7 (41.2)	17 (48.6)	0.769
Extended resection	5 (29.4)	7 (20.6)	0.792
Liver	2 (11.8)	2 (5.9)	
Left adrenal gland	3 (17.6)	3 (8.8)	
Stomach	1 (5.9)	1 (2.9)	
Colon	0 (0)	1 (2.9)	
Portal vein	0 (0)	1 (2.9)	

### Comparison of surgical outcomes for PDAC

Comparison of surgical outcomes of distal pancreatectomy for PDAC is summarized in Table 2. The mean operation time in LDP group and ODP group was similar (190 min vs 245 min, *P* = 0.064). The intraoperative blood loss was significantly lower in LDP group than in ODP group (50 ml vs 400 ml, *P* = 0.000). The first flatus time and diet start time were shorter in LDP group (3d vs 4d, *P* = 0.001; 3d vs 4d, *P* = 0.003). The postoperative length of hospital stay was shorter in LDP group (13d vs 15.5d, *P* = 0.022).

**Table 2:** Comparison of surgical outcomes of distal pancreatectomy for PDAC

Variables	LDP (*n* = 17)	ODP (*n* = 34)	*P* value
Operation time (min)	190 (100–390)	245 (155–420)	0.064
Intraoperative blood loss (mL)	50 (30–500)	400 (100–3900)	0.000
First flatus time (d)	3 (1–4)	4 (2–6)	0.001
First oral intake (d)	3 (1–6)	4 (2–9)	0.003
Pancreatic fistula			0.484
Grade A	6 (35.3)	6 (17.6)	
Grade B	3 (17.6)	9 (26.5)	
Grade C	0 (0)	1 (2.9)	
Clavien-Dindo grade			0.754
Grade I	3 (17.6)	4 (11.8)	
Grade II	3 (17.6)	7 (20.6)	
Grade III	0 (0)	2 (5.9)	
Grade IV	0 (0)	0 (0)	
grade V	0 (0)	1 (2.9)	
Resection margin			0.650
R0	16 (94.1)	29 (85.3)	
R1	1 (5.9)	5 (14.7)	
Postoperative hospital stay (d)	13 (4–23)	15.5 (6–40)	0.022
30 day re-admission	0 (0)	2 (5.9)	0.547

In LDP group 5 patients underwent extended distal pancreatectomy, including resection of stomach in 1 patient, left hepatic lobe in 2 patients and left adrenal gland in 3 patients (in 1 patient both left hepatic lobe and left adrenal gland were resected); while in ODP group, 7 patients had simultaneous resections, including stomach in 1 patient, colon in 1 patient, partial resection portal vein in 1 patient, left hepatic lobe in 2 patients, left adrenal gland in 3 patients (in 1 patient both left hepatic lobe and left adrenal gland were resected). There was one R1 resection in LDP group and five R1 resection in ODP group and showed no significant differences between the two groups (*P* = 0.650).

There were no significant differences in overall postoperative morbidity rate between the two groups (*P* = 0.750). Postoperative pancreatic fistula rates were similar in the two groups (*P* = 0.484) and no C-grade record in LDP group. Only one patient needed reoperation because of intestinal obstruction in ODP group. One death occurred 35 days post-operation during the hospitalization in ODP group. ODP group had 2 (5.9 %) 30 day re-admission because of abdominal infection while LDP group had none (*P* = 0.547).

### Comparison of clinicopathologic characteristics

Comparison of clinicopathologic characteristics of LDP and ODP for PDAC is shown in Table 3. There were no significant differences in tumor sizes (3.5 cm vs 3.9 cm, *P* = 0.664), number of harvested lymph nodes (9 vs 8 *P* = 0.534), ration of N1 (*P* = 0.382), perineural invasion (*P* = 1.000), recurrences (*P* = 1.000). Most patients in both groups had T3 disease. LDP group had 2 (3.9 %) T4 cases. There was no significant difference between the two groups in terms of tumor stage (*P* = 0.090) as well as tumor differentiation (*P* = 0.145). The ration of accepting adjuvant chemotherapy was similar in two groups (*P* = 1.000).

**Table 3:** Comparison of Clinicopathologic Characteristics of distal pancreatectomy for PDAC

Variables	LDP (*n* = 17)	ODP (*n* = 34)	*P* value
Tumor size (cm)	3.5 (2.3–5.5)	3.9 (1.8–5.5)	0.664
Tumor stage			0.090
T1	0 (0)	0 (0)	
T2	3 (17.6)	4 (11.8)	
T3	12 (70.6)	30 (88.2)	
T4	2 (3.9)	0 (0)	
Total LN	9 (5–15)	8 (2–22)	0.534
N1 (positive)	7 (41.2)	19 (55.9)	0.382
Tumor differentitation			0.145
Well	3 (17.6)	7 (23.5)	
Moderate	6 (35.3)	19 (55.9)	
Poor	8 (47.1)	8 (20.6)	
Perineural invasion	12 (70.6)	25 (29.4)	1.000
Adjuvant treatment	13 (76.5)	26 (76.5)	1.000
Recurrences	11 (64.7)	16 (47.1)	0.372

### Survival

**Fig. 1:**
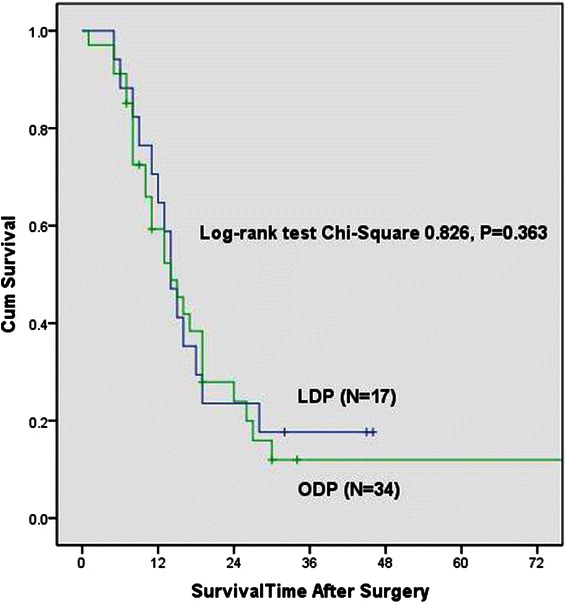
Kaplan-Meier curve of overall survival for LDP and ODP

**Fig. 2:**
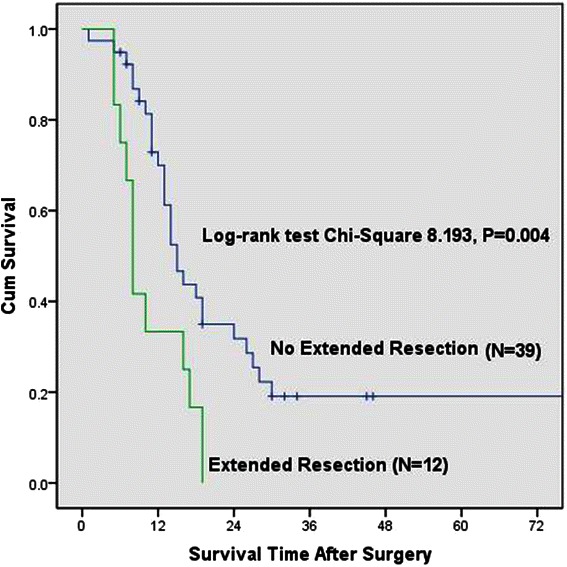
Kaplan-Meier curve of overall survival for extended resection group and no extended group

The mean and median overall survival for the LDP group was 19.9 months and 14.0 months and for ODP group was 22.3 months and 14.0 months. There was no difference in overall survival between the two groups (*P* = 0.802) (Fig. 1). In Cox proportional hazards analysis, tumor size, comorbidity, POPF, tumor stage and adjuvant treatment were not significant for overall survival. Extended resections, R1 resection, perineural invasion and tumor differentitation (Moderate) were associated with worse survival following distal pancreatectomy and the choice of surgical procedure was not associated with the overall survival (Table 4). The mean and median overall survival for group with adjuvant treatment was 27.8 months and 15.0 months while the group without adjuvant treatment was 16.6 months and 13.0 months (*P* = 0.363). The median survival for extended resection group was 8.0 months and for no extended resection group was 15.0 months (*P* = 0.004) (Fig. 2).

**Table 4:** Cox proportional hazards analysis for overall survival

Viables	Hazard ration	95 % CI	*P* value
Adjuvant treatment (no/yes)	0.407	0.207–1.020	0.056
R 1 (negative/positive)		1.260–15.468	0.020
Extended resection (no/yes)		1.105–5.945	0.028
Operation (LDP/ODP)		0.420–1.863	0.885
N1 (negative/positive)		0.745–4.147	0.198
Perineural invasion (negative/positive)		1.240–6.746	0.014
Tumor stage T2	1.000		
Tumor stage T3	0.776	0.292–2.060	0.611
Tumor stage T4	1.174	0.433–3.180	0.753
Tumor differentitation Well	1.000		
Tumor differentitation Moderate	3.432	1.012–11.645	0.048
Tumor differentitation Poor	6.316	0.980–40.700	0.053

*HR* hazard ratio

95 % CI, 95 % confidence interval

## Discussion

In recent years, LDP has been gradually accepted as standard approach to treat benign or low grade lesions located in the body or tail of the pancreas. The technical feasibility, safety and clinical benefit has been well confirmed by various matched studies compared with open distal pancreatectomies [16–18]. However, application of Laparoscopic distal pancreatectomies for PDAC was still limited due to the concern of oncologic outcome and surgical quality [4]. But reports emerging from some experienced centers are encouraging. Compared with conventional open approach, they demonstrated the advantages of less blood loss, shorter hospital stay and early return to normal activity with a similar morbidity, POPF, short oncology outcome (R0 resection rate, the number of harvested lymph nodes) and the overall survival rate [5–9, 19, 20]. In this case-matched study we compared the short-term and long-term outcomes of patients undergoing distal pancreatectomy and the results were consistent with these reports.

Surgery remains the only opportunity for long-term survival for patients with resectable PDAC [21]. R0 resection is the most crucial prognostic factor [22]. In a multicenter analysis Kooby et al. [5] reported that the R0 resection rate of LDP and ODP for PDAC was 73.9 % (17/23) and 65.7 % (46/70). A multivariate analysis was conducted in the whole cohort and only blood >500 ml was associated with R1 resection while the method of resection (LDP or ODP) wasn’t correlated. Shin et al. [20] reported the largest single-institution study of LDP for PDAC (*n* = 70), the R0 resection rate was 75.7 % (53/70) for LDP while 83.8 % (67/80) for ODP. Lee et al. and Hu et al. [8, 9] reported in their series that patients included in LDP group were relatively in early stage and the R0 resection rate was 100 %. These case–control retrospective studies showed no significant difference of R0 resection between the LDP and ODP groups. In the present study, the R0 resections for LDP and ODP were 94.1 % and 85.3 % (*P* = 0.650) which was in accordance with those former studies. Recently, Sharpe et al. [19] reported outcomes for 769 patients of which 144 in the LDP group for PDAC through the National Cancer Data Base. In this retrospective survey, the LDP group had a decrease in margin positivity rate but the tumor size was smaller compared with the ODP group and LDP was more likely to be performed at academic/research institutions. The results were satisfactory for laparoscopic procedure although the heterogeneity might exist due to the type of study design or selection bias. Besides the essentiality of frozen section, extended resections were required in some cases in order for a definitive margin-negative surgery because of the aggressive nature of the disease [23]. Extended resections are feasible procedures with increased postoperative morbidity and better survival compared with palliative bypass procedures [24]. Although laparoscopic extended resection of the pancreas is technically demanding, its application is increasing in specialized centers. Croome et al. [25] reported data from Mayo clinic of 31 patients undergoing total laparoscopic pancreaticoduodenectomy with major vascular resection, there was no significant difference of the total complications comparable with open group and with less mean operative blood, less hospital stay. We previously reported the first laparoscopic hepatopancreatoduodenectomy case with favorable perioperative outcome and showed no sign of recurrence over a year [26]. The data of LDP combined with extended resections is rare. Shin et al. [20] reported 6 (8.6 %) cases of concurrent resections for PDAC by laparoscopic procedure including 5 left colectomies and 1 gastrectomy. After propensity score-matched (including age, BMI, tumor size, concurrent resection) analysis, the overall survival was similar between the LDP group and ODP group while concurrent resection ration were balanced between the two groups. Ricci et al. [27] reported 6 (18.7 %) extended resections including resection of liver wedge, stomach, left adrenal gland and colon among 32 LDP. In our study, we had 5 cases (29.4 %) of extended resections in LDP group with 1 R1 resection while 7 cases (20.6 %) in ODP group with 5 R1 resections. We abolished the laparoscopic procedure of two cases because of invasion to superior mesenteric artery (SMA). Despite the sample data was too small to make any persuasive conclusion, it may achieve R0 resection of locally advanced PDAC in selected patients through laparoscopic procedure by skilled surgeons. Completion of the learning curve, a fixed surgical group and suitable selection criteria were efficacious to carry out these complex goals [27, 28] and we insist on using 5 trocars strategy in order for the cooperation of the main surgeon and the first assistant. Until now, there is no standard indication of LDP for PDAC. As reported from previous studies and meta-analysis, surgeons are mostly inclined to conduct LDP for smaller tumor size [2, 6–8]. Although Kooby et al. [5] reported tumor size (>4 cm) was not associated with positive resection margin, a huge tumor would be an obvious obstacle for exposure of the operation field. So, patients forwith tumor size >5 cm in body and >10 cm in tail of the pancreas were reserved for open procedure and were exluded in this study. The median survival was both 14 months in LDP and ODP groups in this series. Kooby et al. [5] reported median survival 16 months both for LDP and ODP groups and Magge et al. [6] reported 19 months for the entire cohort. Lee et al. [8] reported a median follow-up 39 months for the minimally invasive surgery group (including 4 robotic cases) using their inclusion criteria (Yonsei criteria) which mainly consisted of early stage pancreatic cancer. Compared with previous studies, the survival data in this study was not fulfilling. In Cox proportional hazards analysis extended resection, perineural invasion were strong factors for worse survival. The high ration of extended resection (23.5 %) and perineural invasion (72.5 %) of the whole cohort indicated the cases enrolled in this study were relatively in advanced stage due to lack of early diagnosis of the disease probably. The median survival for no extended resection group was 15.0 months and was consistent with the previous case-matched studies.

This study has several critical limitations, including the retrospective design and low number of patients enrolled in the study. Adjuvant treatment is believed to prolong overall survival [29], but in this study the Cox proportional hazards analysis showed no association with overall survival (*P* = 0.380). The poor differentiation was not associated with overall survival but the moderate differentiation showed association. The small sample of this study might be the reason and didn’t have sufficient statistical power to evaluate the outcome. The study span lasted 11 years and only 1.3 LDP cases per year were performed. The surgical technique was not standardized between the laparoscopic and open approach. Also the follow-up time was short especially for the LDP group and it was difficult to calculate the 5-year survival. Until now, the oncologic safety and long-term survival were not tested by any randomized controlled study between LDP and ODP for PDAC, so it is not sufficient enough to make a conclusion that LDP is oncologic equivalence to ODP [3, 4]. As Kooby and Kang commented it was difficult to conduct an RCT because of the infrequence of diagnosis and opportunity for operation of PDAC in the pancreatic body and tail [4, 5]. The result of this study could provide valuable evidence to support use of LDP for PDAC even in relatively advanced stage.

## Conclusion

In conclusion, the results in our study validated that LDP was technically feasible and safe for PDAC in selected patients and the short-term oncologic outcomes were not inferior to ODP in this small sample study However the long-term oncologic safety of LDP for PDAC has to be further evaluated by multicenter or randomized controlled trials.LDP with extended resection for PDAC is better performed in highly specialized centers and with suitable selection criteria.

## Abbreviations

ASA: American Society of Anesthesiologists
BMI: body mass index
DM: diabetes mellitus
LDP: laparoscopic distal pancreatectomy
ODP: open distal pancreatectomy
PDAC: pancreatic ductal adenocarcinoma
POPF: postoperative pancreatic fistulae
SMA: superior mesenteric artery
